# The need to improve the laws and regulations relevant to the outbreak of COVID-19: What might be learned from China?

**DOI:** 10.7189/jogh.10.010328

**Published:** 2020-06

**Authors:** Hao Li, Mengnan Hu, Shuang Liu

**Affiliations:** 1 Global Health Institute/School of Health Sciences, Wuhan University, Wuhan, China; 2 School of Law, Henan University, Kaifeng, China; 3 Law School, Tianjin University, Tianjin, China; 4 European Law Research Center, Henan University, Kaifeng, China

The novel coronavirus disease (COVID-19) first broke out in Wuhan (China) in December 2019. Then it spread rapidly to whole China and the world [1]. On January 30, the World Health Organization (WHO) declared the outbreak as a public health emergency of international concern [2]. By February 28, 2020, China had reported the highest numbers: 78961 confirmed cases and 2791 deaths. Even after China’s whole society's effort saved the time for other countries for better preparation, the situation has evolved into a pandemic. A total of 51 countries outside of China reported having suspected and confirmed cases. Among them, the Republic of Korea (2337 confirmed cases and 13 deaths), Japan (210 cases and 4 deaths), Iran (245 cases and 26 deaths), Italy (650 confirmed cases and 17 deaths) are suffering most [3]. On February 28, the WHO have assessed and increased the spread risk and impact of the COVID-19 from high to very high at a global level [4].

Current findings from the literature suggest that people infected with COVID-19 show mild symptoms such as fever, cough and hypodynamia. Snuffle, runny nose and diarrhea are found in some infected cases as well. It can further develop into pneumonia or difficulty in breathing [1,5]. Accordingly, the COVID-19 can spread through contact via droplets from cough and sneeze, eating together, etc. It is necessary to get self-quarantined when a person shows some clinical symptoms. They shall contact a hospital for laboratory test confirmation as soon as possible. In this case, they shall avoid social activities so as not to infect others. However, exemptions always happen. People who are suspected or confirmed of the infections may conceal their infections and still behave the same way, interacting with people, causing a lot of unnecessary infections. In fact, this scenario could happen everywhere. Therefore, relevant laws and regulations are urgently required to be in place for better prevention and control the spread of COVID-19.

China has suffered most from the outbreak and has encountered various problems and situations, including people’s intentional concealing of information on their sickness. This in the early stage of the outbreak, has caused a lot of infections to other people, and similar cases have been found in South Korea [6], United Kingdom [7], and other countries. How China addressed this type of problem is very relevant for other countries, so that they could learn and be prepared in advance for better response to potential similar problems that could happen at any time.

## CHINA’S RELEVANT LAWS AND REGULATIONS TO SENTENCE THE CRIMES

As China has the world’s largest population, many crime cases of violating laws and regulations are happening, which are initiated by the suspected and confirmed patients during the COVID-19 outbreak. At first, there was a lack of specific articles to sentence the criminals. However, China has rapidly organized experts to formulate additional relevant legislations for more efficient and effective actions, based on the Chinese Criminal Law. On February 6, China released the Judicial Interpretations on Punishing Legally the Crimes of Violating the Prevention of Novel Coronavirus Infected Pneumonia [8].

According to the Interpretations, if a patient is diagnosed as COVID-19 positive, and he refused to be quarantined for medical treatment, or leave the quarantine area during this period, and enter public places or take public transportations, he/she shall be punished with the crime of “intentional violation of public safety in dangerous ways”. The same punishment applies to a suspected patient when he/she acts in the same way as the above and causes spread of coronavirus. According to Articles 114 and 115 of the Chinese Criminal Law, the defendant shall be punished with the penalty of not less than ten years, life imprisonment or death if the person inflicts serious injury, death on people, or causes heavy losses of public or private property; if the circumstances are minor, he/she shall be sentenced to a fixed-term imprisonment of three to ten years. If the person commits the crime with negligence, he/she shall be punished with the penalty from three years to seven years of imprisonment; if the circumstances are minor, he/she shall be sentenced to a fixed-term imprisonment of not more than three years or a criminal detention for the crime of “negligent violation of public safety in dangerous ways”.

According to Article 330 of the Chinese Criminal Law, a person who violates the Chinese Law on Prevention and Treatment of Infectious Diseases by refusing to execute the prevention and control measures proposed by the health and anti-epidemic authorities, and his/her misconduct has caused the spread of coronavirus or a grave danger of spread, shall be sentenced a fixed-term imprisonment of not more than three years or criminal detention for the crime of “impairing the prevention and treatment of infectious disease”; if the consequences are especially serious, he/she shall be sentenced to a imprisonment of three to seven years.

According to the Chinese Law on Prevention and Treatment of Infectious Diseases, if a unit or an individual violates relevant regulations, refuses the investigations, inspections, sample collection, quarantine and treatment of infectious diseases, and has caused the spread of infectious diseases, civil liability shall be assumed. They shall bear the medical expenses, nursing care, etc. If the infected person died unfortunately or was disabled, the responsible unit or person shall pay the compensations for the death and disability, including mental damage compensations. Besides, in order to protect both sides from potential infections, simple remote video trial procedure can be applied if the defendant agrees.

## IMPLICATIONS TO OTHER COUNTRIES

By March 1, 58 countries outside of China had reported their confirmed cases of COVID-19 [9]. During the outbreak, punishment shall be made in time against the violation of laws and regulations committed by the suspected or confirmed cases. However, many countries may still need to strengthen the specific provisions of their laws and regulations, which should be implemented as soon as possible. For example, in the Italian Criminal Law, the provisions in Article 438 stipulated that, a person causing an epidemic through the spread of germ of pathogen, shall be punished with life imprisonment. According to Article 452, if a person committed the crime negligently, he shall be punished with imprisonment from one year to five years [10]. Both the two articles are interpreted in general and need more detailed explanations.

**Figure:**
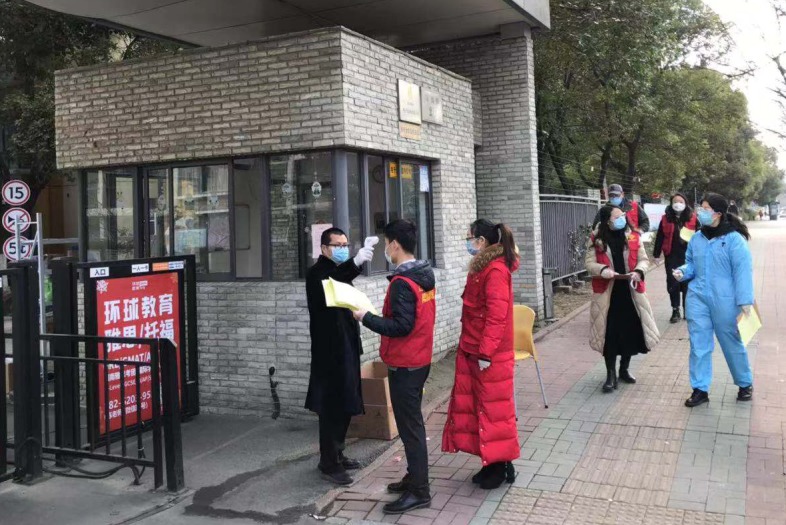
Photo: entry screening in a Chinese community (from the collection of one of the co-authors, used with permission).

Moreover, equal respect, helping reduce suffering and fairness shall be considered as core value during the judgment procedure [11]. Every citizen shall obey the laws and regulations relevant to the prevention and control of novel coronavirus, and responsibility for violating relevant laws and regulations shall be assumed. In order to better improve and implement the epidemic law, the following practices can be learned from China.

1. Preparedness and quick responses are necessary for both government and whole society. It shall be stipulated that all the confirmed and suspected cases have the right to be and shall be quarantined immediately for treatment.
2. It is important to facilitate the process of releasing relevant laws and regulations, and various ways such as TV, social media, etc. shall be in place to notify citizens to increase their knowledge level about relevant laws and the updated regulations as well as the consequences of violating them.
3. As long as a person is identified as violating relevant laws and regulations, legal investigation procedure shall be started immediately whether he is a patient or not. However, if he is a suspected or conformed patient, he can be treated first and punished later.
4. To prevent the spread of coronavirus, remote video trial procedure can be adopted to prevent close contact between the defendant and the judge and other judicial officials.
